# *In Vitro* Inhibition of Angiogenesis by Antibodies Directed against the 37kDa/67kDa Laminin Receptor

**DOI:** 10.1371/journal.pone.0058888

**Published:** 2013-03-12

**Authors:** Raksha Khusal, Bianca Da Costa Dias, Kiashanee Moodley, Clement Penny, Uwe Reusch, Stefan Knackmuss, Melvyn Little, Stefan F. T. Weiss

**Affiliations:** 1 School of Molecular and Cell Biology, University of the Witwatersrand, Johannesburg, Republic of South Africa; 2 Department of Internal Medicine, University of the Witwatersrand, Johannesburg, Republic of South Africa; 3 Affimed Therapeutics AG, Technologiepark, Im Neuenheimer Feld 582, Heidelberg, Germany; The Scripps Research Institute Scripps Florida, United States Of America

## Abstract

The 37kDa/67kDa laminin receptor (LRP/LR) is a central receptor mediating interactions between tumour cells and the basement membrane and is thereby a key player in adhesion and invasion, essential processes in metastatic cancer. To affect continued tumour growth, tumours induce angiogenesis for the constant delivery of nutrients and oxygen. This study aims to determine the blocking effect of the anti-LRP/LR specific antibody, W3 on the angiogenic potential of HUVE (human umbilical vein endothelial) cells. Flow cytometric analysis revealed that 97% of HUVE cells display cell surface LRP/LR. An angiogenesis assay was conducted employing HUVE cells seeded on the basement membrane reconstituent Matrigel™ supplemented with the pro-angiogenic factor vascular endothelial growth factor (VEGF). Post 18h incubation at 37°C tubular structures, namely tube lengths were assessed. Treatment of established tubular structures with 100 µg/ml anti-LRP/LR specific antibody completely blocked angiogenesis. Our findings suggest a central role of the 37kDa/67kDa LRP/LR in tube formation and recommends anti-LRP/LR specific antibodies as potential therapeutic tools for treatment of tumour angiogenesis.

## Introduction

Angiogenesis, the formation of new blood vessels from pre-existing capillaries[1], is a physiologically vital process involved in embryonic development, wound healing; the female menstrual cycle, tissue growth[1] and vascular remodeling.[2] This process is highly regulated in healthy individuals. However, the de-regulation of angiogenesis has been implicated in numerous diseases including rheumatoid arthritis, ischemic heart and limb disease and retinopathy.[1] Angiogenesis is also a vital event in tumour growth and metastasis.[3]

The endothelial cells involved in the angiogenic process are responsive to two sets of cellular signals namely: soluble factors and cell signaling events transduced through the interactions with the extracellular matrix.[4], [5] Soluble pro-angiogenic factors include: basic fibroblast growth factor (bFGF), transforming growth factor-α (TGFα), platelet derived endothelial cell growth factor (PDGF), insulin-like factors (IGF1 and IGF2) and tumour necrosis factor α (TNFα)[6] all of which are constituents of Matrigel™, the basement reconstituent employed in angiogenesis investigations. Furthermore, the vascular endothelial growth factor (VEGF), is the principle angiogenic inducer.[6], [7], [8] Angiogenesis is a multistep process involving endothelial cell activation and subsequent degradation of the surrounding extracellular matrix or basal lamina.[1] This results in protease activation and subsequent release of pro-angiogenic factors/ peptides which in turn stimulate endothelial cell migration towards the angiogenic signal, proliferation and differentiation.[1], [3]

Tumour angiogenesis involves tumour blood vessels that support continued tumour growth.[2] Once tumours exceed a certain maximal diameter, diffusion of oxygen and nutrients become limited and the resultant hypoxia and nutrient deprivation results in the secretion of growth factors and ultimately the onset of angiogenesis and subsequent tumour progression. Thus tumour cells affect vascular endothelial cells by paracrine mechanisms.[9] Owing to the crucial role of angiogenesis in tumour progression and metastasis, selective inhibition of tumour angiogenesis has become a promising approach in anti-cancer therapy.[10]

As previously stated, cell-ECM interactions are imperative in angiogenesis and the basement membrane is of particular importance in this regard. Laminins are cross-shaped trimeric glycoproteins critical in the maintenance of basal membrane structure.[3], [11] Of the 15 available laminin isoforms- laminin-1 (α1β1?1) is of particular interest in angiogenesis as it mediates endothelial cell adhesion and differentiation[1], tube formation and furthermore modulates the activity of endostatin, an angiogenic inhibitor that blocks tube formation[12]. This laminin isoform is the major glycoprotein component of Matrigel™. [3] The α1 chain of laminin-1 contains an IKAV (isoleucine, lysine, alanine and valine) site which promotes collagenase, plasminogen and metalloprotease activity.[3], [13], [14] The activation of these enzymes results in matrix degradation thereby permitting cellular detachment and migration and the release of matrix-sequestered pro-angiogenic factors, all of which are central to successful tube formation.[3]

A central receptor in mediating the cell growth, movement and differentiation properties of laminin is the non-integrin 37kDa/67kDa laminin receptor (LRP/LR) which binds to the ECM component with high affinity.[15], [16] LRP/LR possess two laminin-1 binding sites, a direct binding domain termed a peptide G sequence (161aa–180aa) and an indirect binding domain located towards the carboxyl-terminus (205aa–229aa).[15], [16] This type-II transmembrane receptor is overexpressed in numerous cancers (gastric[17], breast[18], cervical[19], colon[20], colorectal[21], lung[22], ovarian, pancreatic[23] and prostate[24]) , correlates with cancer aggressiveness and it has been proposed that LRP/LR may be indicative of tumour prognosis.[23], [24], [25] LRP/LR downregulation has been shown to induce apoptosis and potentially hamper proliferation in cancer cell lines.[26] LPR/LR is implicated in numerous tumourigenic processes which are akin to angiogenesis namely (tumour) cell adhesion, invasion[27], [28], viability, proliferation and migration.[15], [16] Within classical tumour biology these processes are required for the cell invasion and the formation of metastasis.

Moreover, it is the interaction between LRP/LR and laminin-1 that results in proteolytic activation, a process central to angiogenesis, as previously discussed. Furthermore, a role for LRP/LR in tube formation has previously been proposed.[4] This study aimed to investigate the angiogenic blocking effect of anti-LRP/LR specific antibodies on the *in vitro* angiogenesis of the primary endothelial cell line, human umbilical vein endothelial (HUVE) cells.

## Materials and Methods

### Cell culture and conditions

HUVE cells (Invitrogen, Gibco) were cultured in Medium 200 (Invitrogen, Gibco) supplemented with Low Serum Growth supplement (LSG) (Invitrogen, Gibco) such that the resultant media consisted of: 2% (v/v) fetal bovine serum; 1 µg/ml hydrocortisone; 10 ng/ml human epidermal growth factor (EGF); 3 ng/ml basic fibroblast growth factor (bFGF) and 10 µg/ml heparin.

### Reagents and Antibodies

Matrigel™, employed to induce tube formation is derived from the Engelbreth-Holm-Swarm (EHS) mouse sarcoma, serving as a reconstituted basement membrane, was obtained from BD Biosciences.

Polyclonal anti-LRP/LR antibody W3 was produced as described previously by Rieger et al., (1997). [29]

IgG1-HD37 was recombinantly produced in a mammalian expression system as described by Zuber *et al.,* (2008).[27] In brief, human embryonic kidney cells (HEK293 EBNA) expressing the EBNA-1 gene were transiently co-transfected, by calcium phosphate methodology, with plasmids encoding the heavy (p EU1.2 VH_HD37) and light chains (p EU4.2 VL_HD37) of the anti-Cluster of differentiation 19 (CD19) antibody IgG1-HD37. Affinity chromatography employing protein A sepharose was utilized for antibody purification.

### Indirect Immunofluorescence microscopy

HUVE cells were seeded on sterilised cover slips and upon attaining 30–40%, the culture media was aspirated and cells fixed. Cell surface proteins of interest were detected with the appropriate primary antibodies, anti-LRP/LR specific antibody IgG1-iS18 or anti-cluster of differentiation 31 (CD31) coupled to fluorescein isothiocyanate (FITC) (Sigma Aldrich). These proteins were detected on separate cellular samples. Antibodies were diluted in 0.5% PBS-BSA. Post overnight incubation at 4°C, secondary antibody anti-human-FITC (Beckman Coulter) was added to cells treated with IgG1-iS18 and consequently incubated for 1h (in the dark at room temperature). As the CD31 antibody is a conjugated antibody this step was not performed. Thereafter, cells were subjected to Hoechst 33342 nuclear staining. Fluorescent images were acquired using the Olympus IX71 Immunofluorescence Microscope and Analysis Get It Research Software.

### Flow cytometric Analysis

Flow cytometry was employed to determine LRP/LR levels on the surface of non-permeabilised HUVE cells as described by [28]. Control samples were re-suspended in 100 µl of sheath fluid, whilst the experimental samples were re-suspended in 100 µl anti-LRP/LR specific antibody (IgG1-iS18) solution (30 µg/ml). Post an 1h incubation at room temperature samples were subsequently incubated in the presence of 100 µl anti-human-FITC secondary antibody (20 µg/ml) for 1h. Samples incubated solely with the secondary antibody served to control for background emission and the possible non-specificity of this antibody. Post final incubation, 10 000 cells per sample were analysed employing a Beckman Coulter EPICS® XL-MCL flow cytometer. Data shown is representative of three biological replicates.

### Angiogenesis Assay

To determine the endothelial tube formation potential of HUVE cells and establish the optimal vascular endothelial growth factor (VEGF) concentration required for the induction of HUVE cell tube formation, an angiogenesis assay employing varying VEGF concentrations was conducted. A volume of 50 µl of Matrigel™ (BD Biosciences) was affixed to the wells of a pre-chilled 96 well plate and incubated at 37°C for 1h to allow for Matrigel™ to polymerise. Cell suspensions, in which VEGF (Sigma Aldrich) had been exogenously applied to achieve the varying concentrations (10 ng/ml, 15 ng/ml, 20 ng/ml, 25 ng/ml and 30 ng/ml), were prepared (using Medium 200) and 4×10^4^ cells were seeded in each well. Post incubation at 37°C for 18h, tubular morphology was assessed. A Zeiss inverted microscope was employed to examine tube formation and a Canon Camera V6.0. for imaging the cultures. Remote Capture version 2.7.3.23 and AxioVision LE 4.3 software were used for tube length analysis.

To examine the role of LRP/LR in endothelial tube formation and to evaluate the efficacy of the anti-LRP/LR antibody as an angiogenic inhibitor, an angiogenesis assay (as described above) was performed. Post Matrigel™ preparation, cell suspensions containing 15 ng/ml exogenous VEGF, were employed for cell seeding and post 18h incubation at 37°C, tube length was measured. Conditioned media was gently aspirated so as to minimise tubular disruption, varying antibody concentrations (5 µg/ml, 50 µg/ml and 100 µg/ml) of polyclonal anti-LRP antibody, W3 and IgG1-HD37 (negative control) were composed in Medium 200 and administered to cells. Post 24h incubation at 37°C, cells were again examined and tubular morphology analysed. Comparisons in measurements prior to and post antibody treatment of the same cells were conducted.

### Statistics

Statistical analyses were performed using a two-tailed Students’ *t*-test with a 95% confidence interval. *p-*values < 0.05 were considered significant

## Results

### Human umbilical vein endothelial cells express LRP/LR on their cell surface

**Figure 1:**
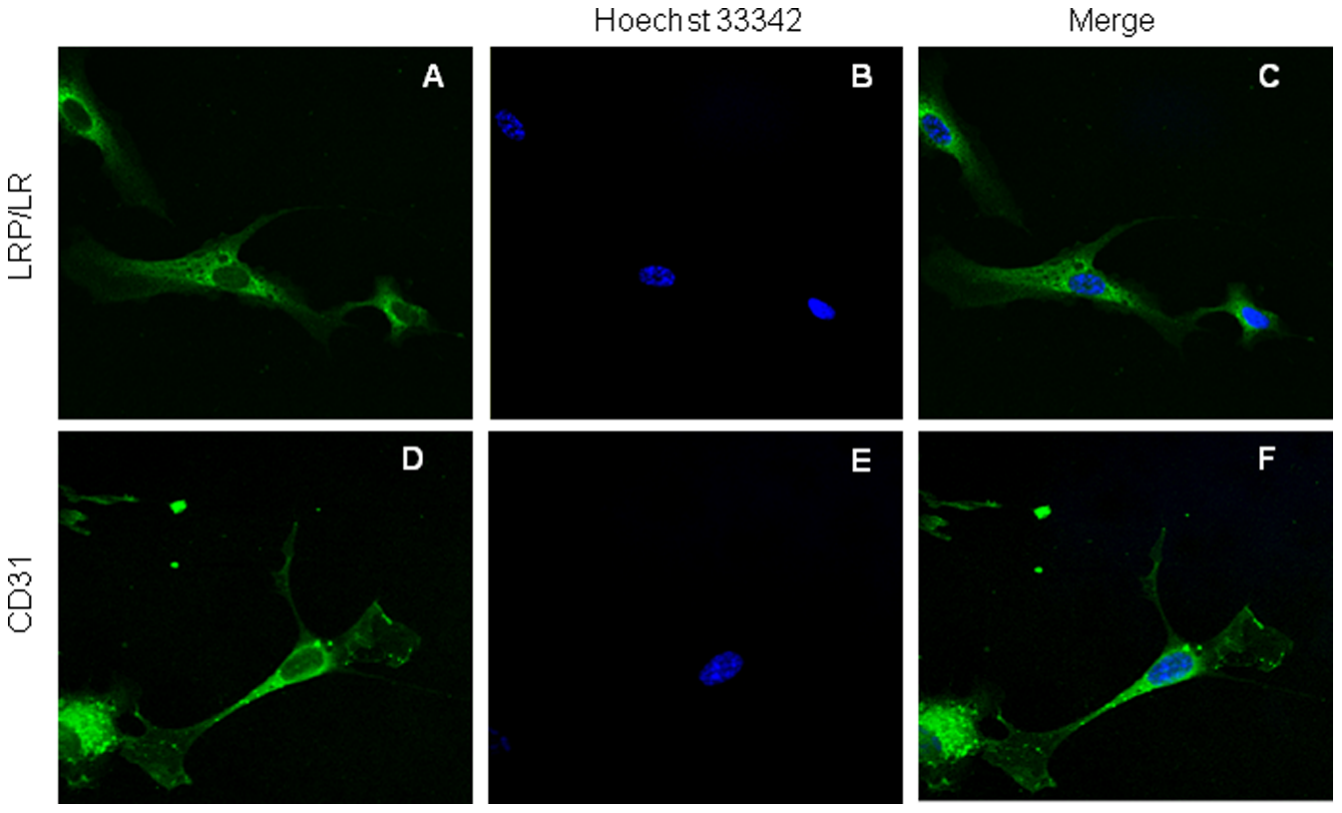
Detection of cell surface 37kDa/ 67kDa LRP/LR and CD31 on HUVE cells by immunofluorescence microscopy. HUVE cells were seeded on coverslips and allowed to proliferate until 30–40% confluency was reached. Non-permeabilised cells were fixed and were indirectly labeled with either an anti-human FITC (fluorescein- isothiocyanate) coupled antibody (Cell Lab) for LRP/LR detection (A) or anti-CD31-FITC antibody (Sigma-Aldrich) (D) . CD31 is an endothelial cell marker and serves as a positive control. Cells were subsequently stained with the Hoechst 33342 nuclear stain (Sigma-Aldrich) (B and E). Merged images (C and F) illustrate cell surface detection of LRP/LR and CD31 in conjunction with nuclear staining, respectively. Magnification: x63. An Olympus IX71 Immunofluorescence Microscope and Analysis Get It Research Software were employed for image acquisition.

**Figure 2:**
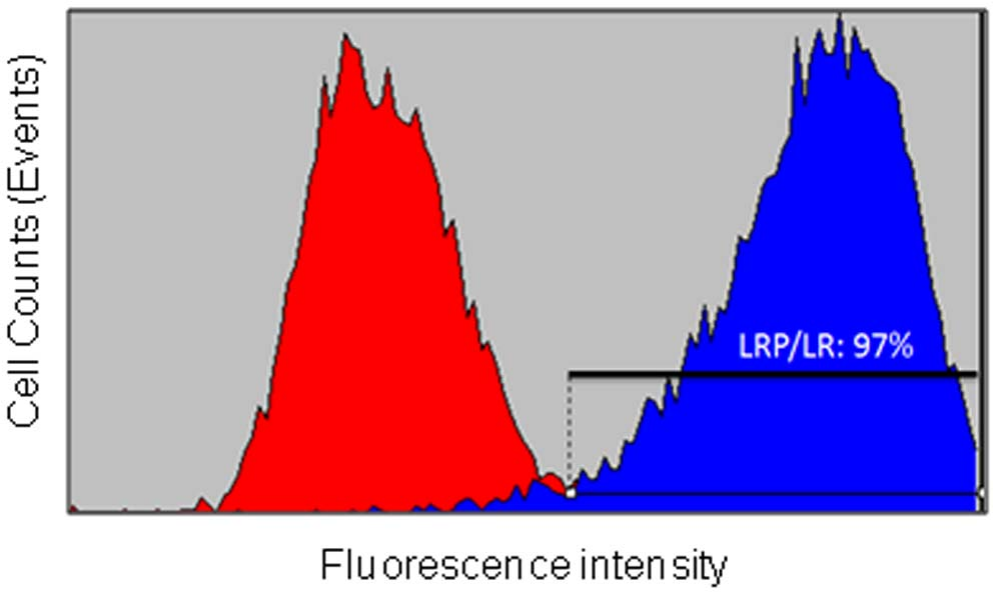
Flow cytometric detection of 37kDa/67kDa LRP/LR levels on the surface of HUVE cells. Cell surface LRP/LR levels on the surface of non-permeabilised HUVE cells were ascertained primarily by incubating cells with IgG1-iS18 followed by incubation with anti-human-FITC coupled secondary antibodies (Sigma-Aldrich). The red curve represents the no antibody control, whilst the blue curve represents treatment with both antibodies. The percentage represents the proportion of cells exhibiting LRP/LR on their cell surface and was calculated using a linked marker from the point of intersection between the curves and the end of the blue curve. A Coulter EPICS® XL-MCL flow cytometer was employed and ten thousand cellular events were counted.

As LRP/LR is a key receptor in mediating cellular adhesion, proliferation and migration, mediating the cellular effects of laminin-1 and has previously been implicated in angiogenesis, we examined whether the receptor was expressed on the surface of the HUVE cell model employed in this study. HUVE cells displayed LRP/LR on their cell surface as is depicted by the positive staining in Fig.1A. Moreover, flow cytometric analysis revealed that 97% of HUVE cells (Fig.2) exhibited LRP/LR on their cell surface further verifying the results obtained by immunofluorescence microscopy. The cluster of differentiation 31 (CD31), also called platelet endothelial cell adhesion molecule (PECAM-1), is an abundantly expressed cell surface marker of endothelial cells involved in wound healing and angiogenesis[30], [31] and served as the positive control (Fig.1D).

### Optimal VEGF concentration for in vitro angiogenesis of HUVE cells

VEGF, the major pro-angiogenic factor, is up-regulated by hypoxia and is a key soluble factor secreted by tumour cells to induce angiogenic processes in endothelial cells (paracrine signaling). Furthermore, VEGF receptors are expressed on endothelial cells such as the HUVE cells but are present on few other cell types. As exogenous VEGF administration is required for tube formation on Matrigel™, we evaluated the concentration of VEGF which would provide maximal angiogenesis, as gauged according to tube length. Statistical evaluation of these results revealed no significant difference between the VEGF treatments (data not shown). However, the 15 ng/ml VEGF treatment displayed the highest average tube length (Table 1) and as such was the concentration employed for subsequent experimentation.

**Table 1:** Effect of varying vascular endothelial growth factor (VEGF) concentrations on *in vitro* HUVE cell angiogenesis.^a^

VEGF concentration (ng/ml)	Average Tube length (µm)
0	12.13
10	12.17
15	13.68
20	10.98
25	12.31
30	10.34

a Cells were seeded on Matrigel™ (BD Biosciences) at a density of 4×10^4^ cells/ well and incubated in 5% CO_2_ humidified atmosphere (37°C) for 18h.

### Anti-LRP/LR specific antibody reverses HUVE cell angiogenesis

**Figure 3:**
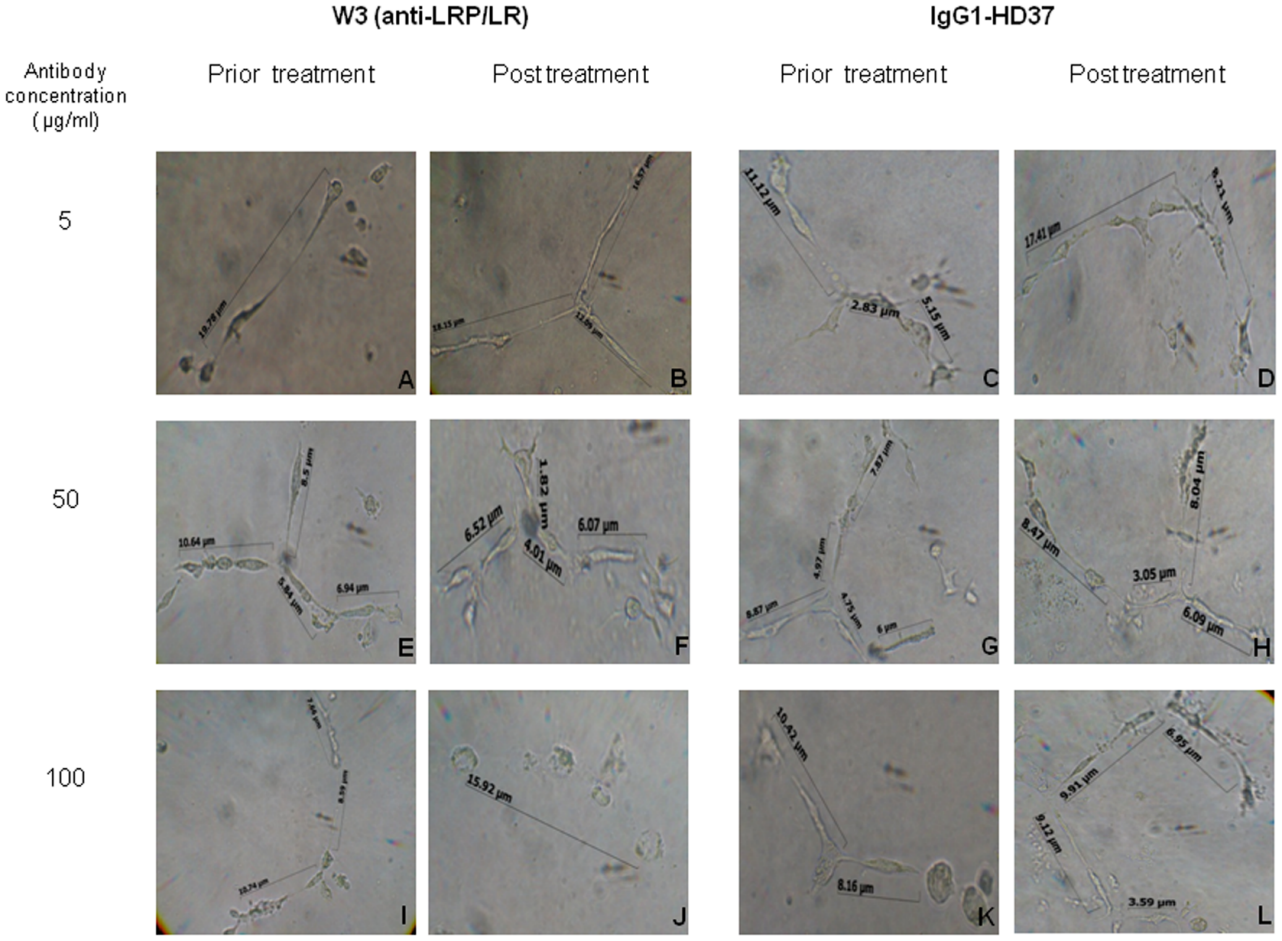
The anti-angiogenic effects of W3 on HUVE cell tube formation. HUVE cell suspensions were prepared with 15 ng/ml exogenously administered VEGF and plated on Matrigel™ (BD Biosciences) at a density of 4×10^4^ cells/ well. Post 18h incubation, tubular structures were microscopically analysed and enumerated by Canon Camera V.6., Remote Capture Version 2.7.3.23 and Axio Vision LE 4.3 software, respectively. Post assessment conditioned media was gently aspirated to ensure minimal disruption of formed tubes, and fresh media with varying concentrations (5 µg/ml, 50 µg/ml and 100 µg/ml) of W3 (B, F, J) or IgG1-HD37 (negative control) (D, H, L) were administered the respective samples. Tubular morphology was assessed (as previously described) 24h post antibody treatment. Magnification: x40

The role of LRP/LR in the induction of angiogenesis has been proposed owing to its close association with tumourigenic processes, its interaction with laminin-1 and its role in the activation of matrix-remodeling enzymes. Thus we investigated whether impedance of the receptor by anti-LRP/LR specific antibody W3 would influence tubular morphology. Treatment of tubular structures with 50 µg/ml of W3 resulted in a significant reduction in tube length of 64.72%, whereas treatment with 100 µg/ml of W3 resulted in a significant 100% reduction in tube length (Fig.3 and Table 2). Treatment of tubular structures with IgG1 HD37 directed against CD19 did not significantly reduce tube length (Fig.3 and Table 2).

**Table 2:** Percentage reduction^a^ of endothelial tube length in HUVE cells.

		W3		IgG1-HD37	
		Percentage reduction in tube length (%)	*p-*value	Percentage reduction in tube length (%)	*p-*value
Antibody concentration (µg/ml)	5	–21.62^b^	0.2980	1.85	0.9674
	50	64.72	0.0082	50.87	0.243
	100	100	0.0024	40.10	0.0544

a Reductions are calculated based on comparisons between the tube lengths of antibody treatments and no antibody treatments. Average tube length of the “No antibody” treatment was set to 100%.

b The negative value is indicative that the average tube length was 21.62% greater than that on the “ No antibody” treatment (therefore 121.62%) and therefore rather than a tube reduction an increase was observed.

## Discussion

Angiogenesis has received considerable attention over the past few decades as a possible target for pathological diseases which require vascularisation, most notably cancer.[9] Through selective inhibition of tumour angiogenesis, tumour growth and progression and the success of metastatic tumourigenic cells at distal sites, owing to oxygen and nutrient deprivation, will be halted. Thus therapeutics aimed at decreasing vascularisation are promising anti-cancer tools which may be effective against numerous cancers.

The rate-limiting step in the angiogenic process is the degradation of the basement membrane which is promptly followed by endothelial cell detachment, proliferation and re-organisation into tubular structures. A key receptor in cellular adhesion to the basal membrane is the 37kDa/67kDa LRP/LR.[28] Through interactions with the laminin-1, the major glycoprotein component of the basal lamina and Matrigel™ basal membrane reconstituent employed here, LRP/LR mediates cellular attachment and induces proteolytic activation of type IV collagenase and other matrix metalloproteases.[32], [33] These in turn degrade the basal membrane, release matrix-sequestered pro-angiogenic factors and allow for cellular migration towards the angiogenic stimulus. Thus, since angiogenesis requires basal membrane degradation and LRP/LR plays a fundamental role in this process, immunofluorescence microscopy and flow cytometry analyses were performed to detect and determine the proportion of HUVE cells which expressed LPR/LR on their cell surface. Once LRP/LR was confirmed to be located on the cell surface of HUVE cells (Fig.1A), flow cytometric analysis revealed that 97% of the examined cells displayed LRP/LR on their cell surface (Fig.2). It has been reported that neoplastic cell lines express very high levels of LRP/LR on their cellular surface when compared to non-tumorigenic controls[27], [28] and that these elevated levels correlate with an increased invasive potential.[27], [28] Although HUVE cells are non-tumorigenic, the high LRP/LR levels correlates to the invasive role of these cells as they are required to degrade the basal membrane and migrate towards stimuli for the formation of 3D tubular structures.

Thus far, the most influential inducer of angiogenic activity is the stimulation of the VEGF molecular signaling pathway.[34] It has been reported that successful angiogenesis may be induced upon administration of VEGF within the 10 ng/ml – 30 ng/ml range.[35], [36], [37] However, the exogenous administration of VEGF has been shown to possess a biphasic response.[38] In this study, maximal tube length was observed at a VEGF concentration of 15 ng/ml (Table 1). Therefore, the application of 15 ng/ml exogenous VEGF in subsequent experiments was justified.

Previous studies have shown that the adhesive and invasive potential of numerous cancer types (fibrosarcoma, lung, cervical, breast, colon and prostate) is significantly reduced upon application of anti-LRP/LR specific antibodies, namely IgG1-iS18.[27], [28] Other tools targeting LRP/LR, including RNA interference (RNAi) technology, the pentosan polysulfate and the heparan mimetic HM2602[16], [27], [28] have similarly hampered the invasion of tumourigenic cells. The mechanism of action whereby these modalities are suggested to impede invasion is through the impedance of the LRP/LR – laminin-1 interaction which subsequently thwarts cellular adhesion, this being a vital process preceding cellular invasion.

**Figure 4:**
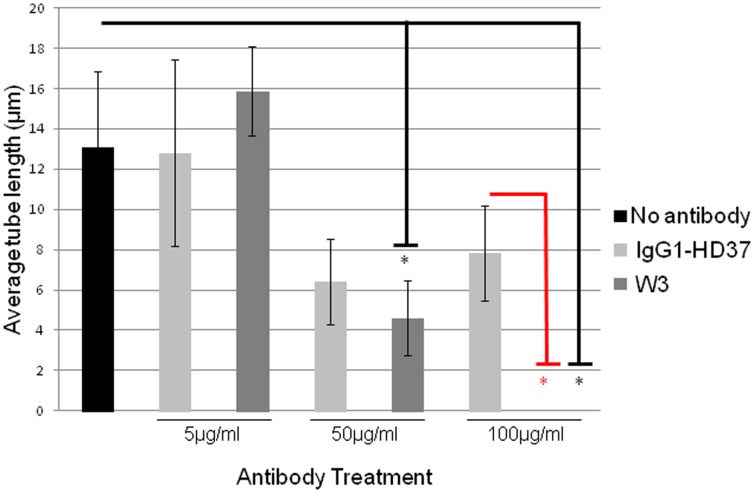
The effects of antibody treatment on the average tube length of HUVE cells. HUVE cell suspensions containing 15 ng/ml VEGF were prepared and plated on Matrigel™ as previously described. Post treatment with varying concentrations of (5 µg/ml, 50 µg/ml and 100 µg/ml) W3 or IgG1-HD37, tube length was enumerated. The bar graph depicts the average tube length post treatment. Error bars represent sd. *p<0.05; Student’s *t*-test.

**Figure 5:**
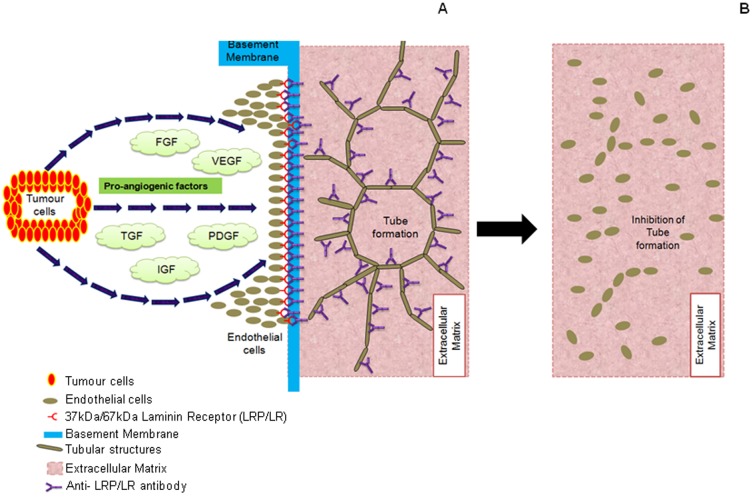
A schematic representation of the effect of anti-LRP/LR specific antibodies on angiogenic tube formation. (A) The administration of anti-LRP/LR antibody W3, to HUVE cells which had established tubular structures on Matrigel^™^, inhibited further degradation of the basement membrane, a requirement for tube formation. This halted the development for additional tubular structures. Moreover, the antibody also bound to existing tubes and thereby blocked the interaction between LRP/LR and Laminin-1, hence resulting in (B) the reversal of tube formation and cells were consequently observed as single cells on the Matrigel^™^.

HUVE cell angiogenesis was similarly disrupted (50 µg/ml) (Fig. 3F) and completely abolished (100 µg/ml) (Fig.3J) upon administration of the anti-LRP/LR specific antibody. When compared to the no antibody control, a significant tube length reduction of 64.72% and 100% was observed upon treatment with 50 µg/ml and 100 µg/ml W3, respectively (Fig.4 and Table 2). These results therefore demonstrate that anti-LRP/LR specific antibody W3 significantly blocked tube formation by HUVE cells – thereby reiterating the fundamental role of LRP/LR in angiogenesis. This is depicted schematically in Fig.5. This is the first work to demonstrate that antibodies directed against the non-integrin laminin receptor (LRP/LR) may inhibit the morphogenesis of endothelial cells into tubular structures. It has also been reported that antibodies directed against laminin-1 under similar experimental conditions (HUVE cell induced angiogenesis on Matrigel™), did not inhibit cellular adhesion to the matrix but did preclude tube formation.[39] Therefore, it may be suggested that the anti-LRP/LR antibody W3, blocked the interaction between LRP/LR and laminin-1, thereby ceasing differentiation of HUVE cells into tubular structures.

In summary, the strikingly significant abolishment of tubular structures in the HUVE cell angiogenesis model by W3, suggests that anti-LRP/LR specific antibodies may prove a potential therapeutic tool for the treatment of tumour angiogenesis.
